# Mesenteric venous thrombosis: a rare complication of small bowel neuroendocrine tumor presenting with gangrenous appendicitis

**DOI:** 10.1093/jscr/rjac092

**Published:** 2022-03-22

**Authors:** Arshad M Bachelani

**Affiliations:** Department of Surgery, Penn Highlands Mon Valley, Monongahela, PA 15063, USA

## Abstract

A 61-year-old woman presented to the hospital with a clinical picture consistent with acute appendicitis. During surgery, the appendix was found to be gangrenous and involved the appendiceal base, so an ileocecectomy was performed. Pathology revealed an incidental neuroendocrine tumor of the terminal ileum involving five of nine lymph nodes. The patient later developed mesenteric venous thrombosis but was diagnosed and treated promptly, and she is now doing well. There have been previous reports of small bowel neuroendocrine tumor resulting in bowel ischemia, usually due to fibrosis which can result in obstruction of the mesenteric vessels. However, this is the first known report of a small bowel neuroendocrine tumor presenting with appendicitis, which most likely was from an ischemic etiology. This case also demonstrates the importance of a high index of suspicion for mesenteric ischemia in patients with small bowel neuroendocrine tumor who present with acute abdominal pain.

## INTRODUCTION

Small bowel neuroendocrine tumors are rare, and symptoms are often vague [1]. Unfortunately, many patients already have metastatic disease by the time a diagnosis has been made. These tumors may induce a fibrotic reaction that can lead to bowel obstruction, but it can also lead to venous stasis with resultant bowel ischemia [2]. This article describes a patient with small bowel neuroendocrine tumor who initially presented with gangrenous appendicitis and then developed acute mesenteric venous thrombosis.

## CASE PRESENTATION

The patient is a 61-year-old female who presented to the hospital with a 2-day history of right lower quadrant abdominal pain. She had a history of hypertension, hypercholesterolemia and a hysterectomy but was otherwise healthy. Her body mass index was 31.6 kg/m^2^. She was a non-smoker and had a family history of breast cancer and heart disease. She was afebrile and hemodynamically stable. One examination, she had bilateral lower quadrant abdominal tenderness, right greater than left. Workup revealed a white blood cell count of 13.7 thousand and a lactic acid of 1.5 mmol/l. Computed tomography (CT) scan of the abdomen and pelvis revealed an inflamed appendix with mesenteric fat stranding, consistent with acute appendicitis.

**Figure 1:**
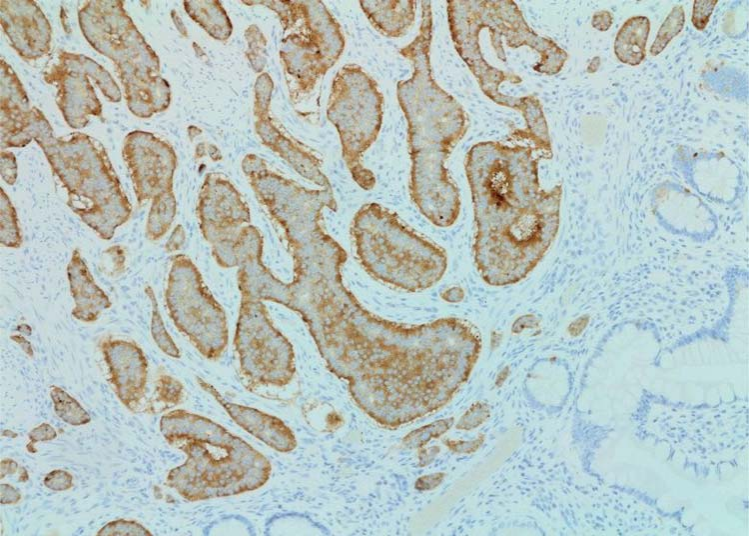
Synaptophysin stain (×100 magnification) of small bowel neuroendocrine tumor.

**Figure 2:**
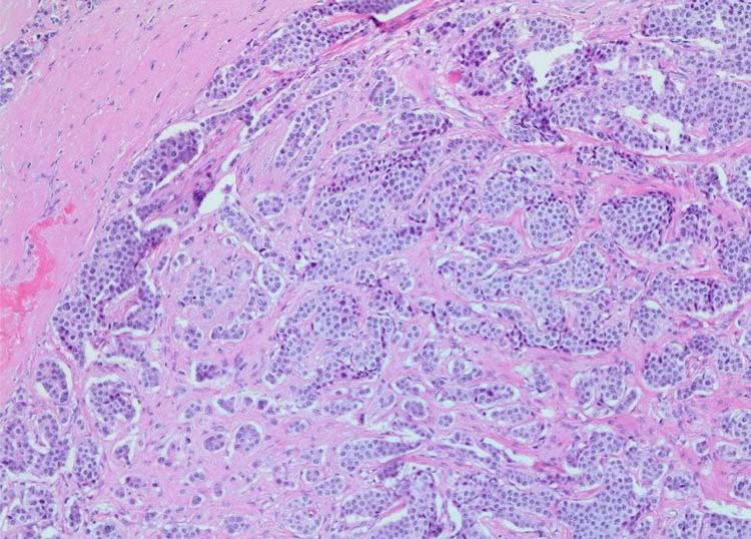
Subtotal lymph node replacement by tumor (×100 magnification).

The patient was taken to the operating room for a laparoscopic appendectomy. During surgery, the appendix was noted to be gangrenous and perforated. Because the inflammation involved the entire appendix including the appendiceal base, decision was made to perform an ileocecectomy. Pathology revealed a gangrenous perforated appendix with periappendiceal abscess along with a well-differentiated, 1.2 cm neuroendocrine tumor of the terminal ileum (Figs 1 and 2). Mitotic index was 2.8 mitoses per 2 mm^2^, and Ki67 proliferative index was 3%. Five of nine lymph nodes were positive for metastatic neuroendocrine tumor with extranodal extension of the tumor and perineural invasion. Surgical margins were clear. Tumor, node and metastasis (TNM) histopathologic stage was pT2, N1. The patient developed a postoperative ileus but this resolved and she was discharged on postoperative day 7.

**Figure 3:**
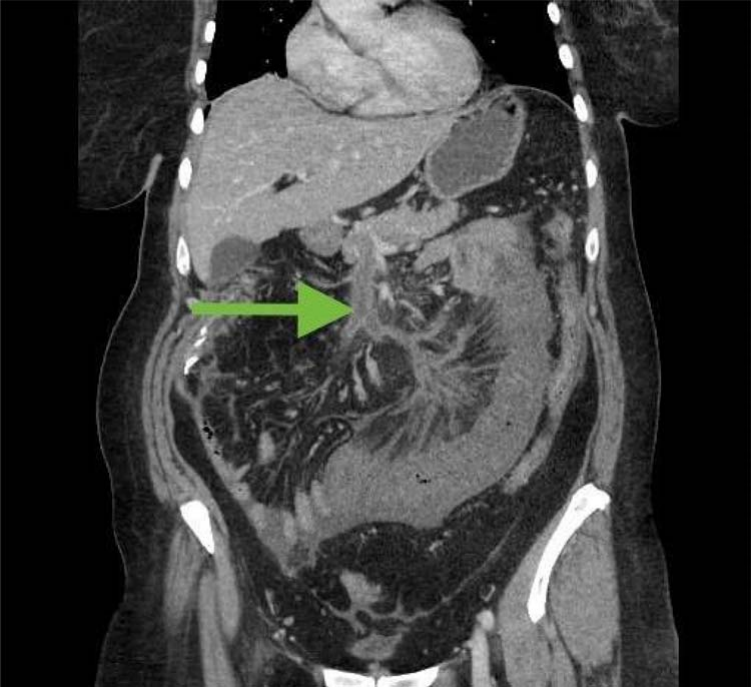
CT abdomen/pelvis 2 weeks after initial surgery demonstrating superior mesenteric vein thrombosis (arrow) and a loop of thickened small bowel consistent with ischemia.

Two weeks after discharge, the patient returned with crampy abdominal pain. She was moderately tender on examination. White blood cell count was 15 000 and lactic acid was elevated at 3.5 mmol/l. CT scan of the abdomen and pelvis revealed an intraluminal filling defect of the superior mesenteric vein and portal vein not present on prior CT, consistent with acute venous thrombus (Fig. 3). There was also a long segment of concentric wall thickening of small bowel with mesenteric edema. The patient was taken to the operating room and had a segment of ischemic small bowel resected. She was also started on anticoagulation. Her abdomen was left open and she was taken back to the operating room a couple days later, at which time an anastomosis was created and her abdomen was closed.

After discharge, the patient had a PET scan that was normal. Her urinary 5-HIAA was 2.1 mg/24 hours, and her chromogranin A levels have remained normal. She was not placed on any adjuvant treatment. It has now been 2 years since she first presented and she is doing quite well.

## DISCUSSION

**Figure 4:**
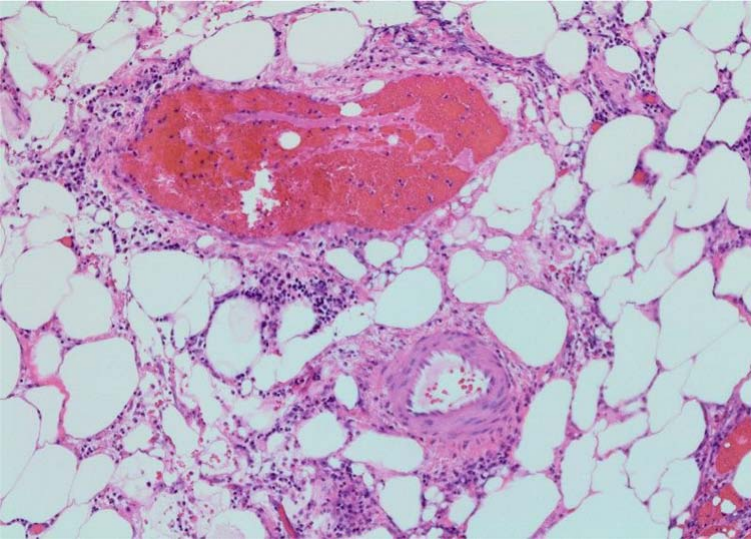
Small vein thrombosis, from initial operation (×100 magnification).

Neuroendocrine tumors are a rare, heterogeneous group of neoplasms. The incidence is about one in 100 000, although this may be increasing [3]. These tumors grow slowly and symptoms are often vague, which may lead to a delay in diagnosis. The primary treatment for small bowel neuroendocrine tumors in patients with locoregional disease is surgical resection with lymphadenectomy, although there are now several treatment options for distant metastases [4]. Metastases to the mesentery are common, and these metastases with released cytokines can lead to fibrosis. This can result in a bowel obstruction or ischemia. In one retrospective study looking at 824 patients with small bowel neuroendocrine tumors, 36 patients had apparent clinical manifestations of fibrosis, which included mesenteric vessel obstruction and obstructive uropathy [2]. There has been one reported case of cecal necrosis as a result of small bowel neuroendocrine tumor [5]. However, this is the first case of small bowel neuroendocrine tumor presenting with gangrenous appendicitis. In our patient, the patient was found to have an incidental neuroendocrine tumor of the terminal ileum with mesenteric metastases and extranodal extension. This likely caused ischemia of the appendix. In retrospective review of pathology slides from the initial operation, the patient did have small vein thrombosis present (Fig. 4), although this is not necessarily uncommon in appendicitis. From a surgical perspective, the entire appendix was inflamed, whereas most patients with appendicitis have a healthy base that can be safely divided during appendectomy. Perhaps fortuitously, this led us to proceed with ileocecectomy, which resulted in her ultimate diagnosis.

The classic teaching regarding the pathophysiology of acute appendicitis has been an etiology of luminal obstruction, which leads to increased intraluminal pressure. This leads to progressive inflammation, dilation and subsequent perforation. However, recent studies have questioned whether this sequence is necessarily true in all cases. There could potentially be multiple possible etiologies for acute appendicitis, including ischemic and neuroimmune mechanisms [6]. In our patient, the acute appendicitis likely stemmed from an ischemic etiology.

Mesenteric venous thrombosis has been reported in patients with neuroendocrine tumors, either from direct encasement of vessels which leads to venous stasis or due to the cytokine response that leads to fibrosis. In the literature, there have also been several reports of mesenteric venous thrombosis as a result of acute appendicitis itself [7, 8]. It may be impossible to tell which of these was the prominent factor for our patient. Regardless, she likely had the classic picture of Virchow’s triad, including venous stasis, hypercoagulability from her tumor and endothelial injury. Mesenteric venous thrombosis is a serious condition that can quickly lead to intestinal ischemia and has a mortality of up to 20% [9]. Treatment primarily consists of anticoagulation and surgical resection of any ischemic segments of bowel, although options can also include thrombectomy and catheter-directed thrombolysis [10].

## CONCLUSION

The symptoms of small bowel neuroendocrine tumors can be quite vague; this can lead to a delay in diagnosis, although many are found incidentally. A high index of suspicion for mesenteric ischemia should be held for patients with small bowel neuroendocrine tumor who present with acute abdominal pain.

## CONFLICT OF INTEREST STATEMENT

None declared.

## FUNDING

None.
